# Bio-Inspired Take-Off Maneuver and Control in Vertical Jumping for Quadruped Robot with Manipulator

**DOI:** 10.3390/mi12101189

**Published:** 2021-09-30

**Authors:** Ru Kang, Fei Meng, Lei Wang, Xuechao Chen, Zhangguo Yu, Xuxiao Fan, Ryuki Sato, Aiguo Ming, Qiang Huang

**Affiliations:** 1 Intelligent Robotics Institute, School of Mechatronical Engineering, Beijing Institute of Technology, Beijing 100081, China; kangru@bit.edu.cn (R.K.); wanglei_bit@bit.edu.cn (L.W.); chenxuechao@bit.edu.cn (X.C.); yuzg@bit.edu.cn (Z.Y.); yuyan0672@163.com (X.F.); ming@mce.uec.ac.jp (A.M.); qhuang@bit.edu.cn (Q.H.); 2 The Beijing Advanced Innovation Center for Intelligent Robots and Systems, Beijing Institute of Technology, Beijing 100081, China; 3 Department of Mechanical Engineering and Intelligent Systems, The University of Electro Communications, Tokyo 182-8585, Japan; sato_r@rm.mce.uec.ac.jp

**Keywords:** quadruped robot, vertical jumping, take-off maneuvers, manipulator

## Abstract

The jumping motion of legged robots is an effective way to overcome obstacles in the rugged microgravity planetary exploration environment. At the same time, a quadruped robot with a manipulator can achieve operational tasks during movement, which is more practical. However, the additional manipulator will restrict the jumping ability of the quadruped robot due to the increase in the weight of the system, and more active degrees of freedom will increase the control complexity. To improve the jumping height of a quadruped robot with a manipulator, a bio-inspired take-off maneuver based on the coordination of upper and lower limbs is proposed in this paper. The kinetic energy and potential energy of the system are increased by driving the manipulator-end (ME) to swing upward, and the torso driven by the legs will delay reaching the required peak speed due to the additional load caused by the accelerated ME. When the acceleration of ME is less than zero, it will pull the body upward, which reduces the peak power of the leg joints. Therefore, the jumping ability of the system is improved. To realize continuous and stable jumping, a control framework based on whole-body control was established, in which the quadruped robot with a manipulator was a simplified floating seven-link model, and the hierarchical optimization was used to solve the target joint torques. This method greatly simplifies the dynamic model and is convenient for calculation. Finally, the jumping simulations in different gravity environments and a 15° slope were performed. The jump heights have all been improved after adding the arm swing, which verified the superiority of the bio-inspired take-off maneuver proposed in this paper. Furthermore, the stability of the jumping control method was testified by the continuous and stable jumping.

## 1. Introduction

The design of a bionic robot benefits from the introduction of robust and energy-saving motion based on animal motion, which gives it huge application potential in challenging artificial and natural environments. Compared with wheeled or tracked vehicles, quadruped robots have significant advantages in rugged or unstructured terrain. Especially in planetary exploration missions such as microgravity celestial bodies, jumping motion enables legged robots to overcome obstacles and achieve effective and rapid motion [1]. The German Research Center for Artificial Intelligence developed the four-legged Mantis robot [2], which can use two of its six limbs for manipulation. Furthermore, the robot is capable of walking on all six extremities, which is a big advantage in difficult terrain. Lehner [3] proposed a system architecture for an autonomous rover for planetary exploration, the light weight rover can lift the scientific instrument from the ground and places it into the payload carrier on the robot’s back. The SpaceBok [4] robot equipped with adaptive planar feet achieved locomotion walking up a 25° inclined Mars analog slope. The research above reflects the superiority of four-legged mobile platforms in planetary exploration. Quadruped robots have recently demonstrated impressive advanced dynamic motion capabilities using a variety of driving methods and control strategies, such as BigDog [5], MIT Cheetah3 [6], SpotMini [7], ANYmal [8], HyQ [9], Ghost [10] and Aliengo [11]. Typical quadruped robot tasks include patrolling in challenging terrain and inspecting scenes in space. However, direct interaction with the environment is limited to the contact of movement, and there is little flexibility in the operation ability. A quadruped robot equipped with a multi-degrees of freedom (DoFs) manipulator can expand the way it interacts with the real world. Such a robot will be able to carry and move objects, help people transport payloads, open doors and interact with the surrounding environment in a way that was previously impossible.

The quadruped robots with a manipulator have gradually become a research hotspot. A robot with high DoFs and dynamic control of heavy objects was proposed [12]. By studying the coordinated use of the body, legs and integrated manipulator on the mobile robot, an excellent dynamic throwing experiment was realized. A multi-legged mobile robotic manipulator was developed based on HyQ, which integrates the mobile platform controller and robust arm controller and combines the control framework of a payload estimation module [13]. SpotMini [7] has achieved impressive results, which can demonstrate operational tasks when walking, such as opening a door and carrying a payload. The motion planning and control framework ALMA for torque-controlled quadruped robots was proposed [14], which can perform dynamic motion while performing operation tasks. A unified model predictive control (MPC) framework was proposed, which plans the whole body motion/force trajectory task and combines dynamic motion and manipulation. Additionally, the robustness to model mismatch and external interference was verified by pushing/pulling a heavy resistance gate [15]. The above-mentioned quadruped robot with a manipulator has relatively stable coordinated motion and simple operation behavior, and it does not show dynamic motion ability. A legged robot has significant advantages in rugged terrain and high obstacles environments, and dynamic jumping ability is its key performance requirement. Pavei [16] have also demonstrated that bouncing gaits benefit more in low gravity than walking and that skipping reports the highest gain in cost reduction, reaching values for terrestrial walking.

There is some research focused on the obstacle crossing and jumping control of a quadruped robot. MIT Cheetah 3 can jump up the table (0.76 m); the main technical approaches include effective trajectory optimization and accurate high-frequency tracking controller [17]. A planning framework for a quadruped robot was proposed to cross obstacles, which combines the use of heterogeneous simplified models on different prediction time scales. MIT Cheetah 2 can automatically jump over obstacles as high as 40 cm [18]. The parallel elastic legs were proposed to provide temporary storage and reuse energy during jumping, and jumping with integrated elasticity significantly reduced energy consumption through experimental analysis [1]. A framework of deep reinforcement learning was proposed, and its robustness was verified in the simulation environment [19]. The dynamic motion of the above was achieved through trajectory optimization or reinforcement learning, and the stable dynamic jump of the quadruped robot was realized by a robust landing controller. The introduction of a manipulator will bring new challenges to the dynamic jumping motion for the robot; the increase in the system’s mass will result in a decrease in the jumping height, and more active DoFs will increase the control complexity.

However, there is no research on using additional manipulators to help the robot jump only on manipulators as a burden. In order to overcome the above-mentioned challenges brought by the addition of manipulators, a quadruped robot with a manipulator was developed in this paper. We selected the symmetrical structure leg in which the two joints can provide equal contributions to the vertical movement. At the same time, a jump control framework based on a whole-body controller (WBC) was established, which includes a bio-inspired take-off maneuver and the whole-body motion control based on hierarchical optimization to realize continuous and stable jumping for our quadruped robot with a manipulator. The main contributions of this paper are as follows:

(1) To improve the jumping height of a quadruped robot with a manipulator, a bio-inspired take-off maneuver based on the coordination of upper and lower limbs was proposed in this paper. The kinetic energy and potential energy of the system are increased by driving the manipulator-end (ME) to swing upward, and the torso driven by the legs will delay reaching the required peak speed due to the additional load caused by the accelerated ME. When the acceleration of ME is less than zero, it will pull the body upward, which reduces the peak power of the leg joints. Therefore, the jumping ability of the system is improved. Furthermore, the optimal jumping planning was obtained by optimizing the trigger time.
(2) To realize continuous and stable jumping, a control framework based on whole-body control was established, in which the quadruped robot with a manipulator was simplified into a floating seven-link model, and the hierarchical optimization method was used to solve the target joint torques. This method greatly simplifies the dynamic model and is convenient for calculation.

This paper is organized as follows. Section 2 introduces the system overview. In Section 3, the bio-inspired take-off maneuver is proposed. The motion tracking based on whole-body control is described in Section 4. Numerical simulations and discussions are introduced in Section 5. The last Section summarizes this paper and proposes future work.

## 2. System Overview

### 2.1. Leg Structure Selection

**Figure 1:**
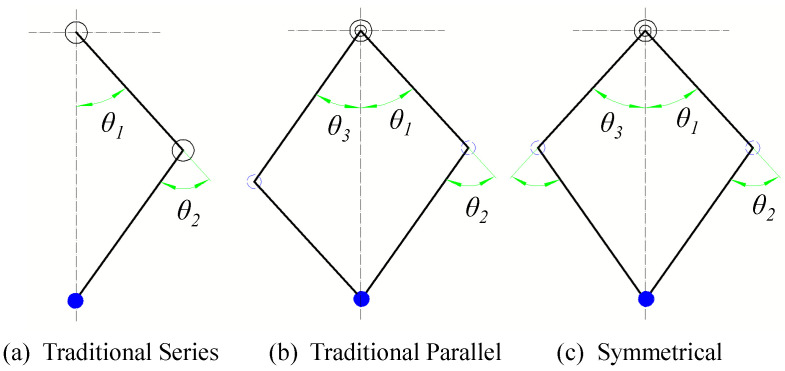
Three leg structures: (**a**) Traditional series (TS), (**b**) traditional parallel (TP) and (**c**) symmetrical leg (SY).

The leg mechanism plays an important role in the kinematic and dynamic characteristics of robots. We have given the definition of different legs using the example of a single leg with two DoFs in detail [20]. As shown in Figure 1a–c, the leg rod driving modes of different types are distinct. To compare the load capacity of the candidate leg, we calculate the required torque of the two driving joints under the same leg length configuration and external force. By deriving the forward kinematics of different structures, their foot positions can be expressed uniformly using the joint angle and length of leg rods; the relationship between the joint torque **τ** and the foot-end force ***f*** can be expressed uniformly, as shown in Equation (1):

(1) $$\dot{P}=J\left(\Theta \right)\dot{\Theta },\;\tau \;=\;J{\left(\Theta \right)}^{T}f,$$

where $\dot{P}$ represents the foot-end velocity in the Cartesian coordinate, $\dot{\Theta }$ represents the joint angle velocity, and J(Θ) is the Jacobian matrix.

As shown in Figure 1, the angle of the driving link must be taken as a variable when solving the Jacobian matrix J(Θ). Furthermore, the driving joints of the three leg structures are different, so the Jacobian matrix for solving the torque is different. In the TS structures shown in Figure 1a, the hip joint angle and knee joint angle are both controlled directly by their respective motors and the angular variables are $\theta_{1}$ and $\theta_{2}$. The knee joint of the TP structure is not driven directly by the knee motor, as shown in Figure 1b, but are coupling-driven by the hip and knee joint motor. The angular variables are $\theta_{1}$ and $\theta_{3}$ = ($\theta_{1}$ + $\theta_{2}$). Because of the structural characteristics of the SY leg, the relationship between the joint angle and the driving motor angle is more complex. The angular variables are $\theta_{1}$ and $\theta_{3}$. The detailed derivation was shown in [20]. In order to compare the bearing capacity of the three structures more intuitively, we choose the same workspace as shown in Equation (2):

(2) $$-\pi \leq \theta_{1}\leq \pi ,-2\pi \leq \theta_{2}\leq 0,-\pi \leq \theta_{3}\leq \pi$$

where the definition of $\theta_{1}$, $\theta_{2}$ and $\theta_{3}$ was shown in Figure 1.

Through comparative analysis, the reachable workspace of the series leg is the largest, and this advantage is only reflected in some demonstrative exercises of simulated gymnastics. Furthermore, the workspace of TP and SY is enough for most of the basic actions. The calculations indicate that the SY structure has the largest load capacity in the same workspace, it also has the more superior joint motion performance [20], which makes it more suitable for a loaded robot.

In the microgravity working environment, such as planet exploration, jumping is an important dynamic motion mode. As we all know, the jump height is related to the peak speed and peak torque in the vertical jumping movement, but they need not reach the peak at the same time. Therefore, this paper analyzed the instantaneous power comparison in the same jump process for three candidate structures based on the previous research, which can be calculated by Equation (3):

(3) $$P_{j}=\tau_{j}\omega_{j}$$

where $\tau_{j}$ and $\omega_{j}$ is the torque and angle velocity of the joint, respectively.

**Figure 2:**
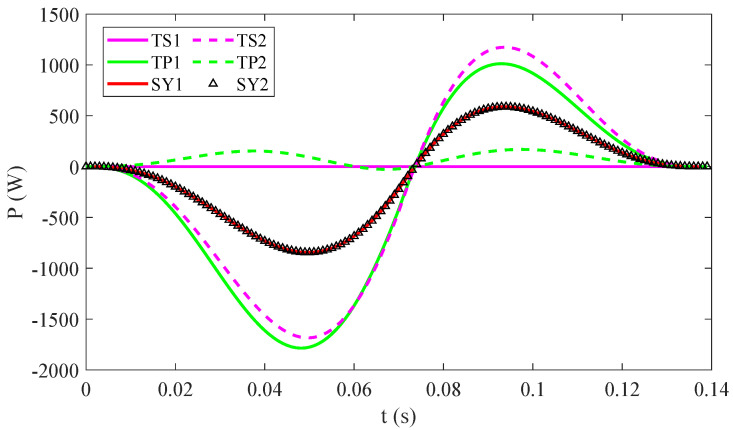
The required power curves of each joint during vertical jumping.

We calculated the required joints’s powers of the three legs during the same motion (vertical jump within 0.14 s) and assumed that the leg rods have no mass. As shown in Figure 2, the maximum instantaneous powers required by joint TS2 and TP1 are 1685 W and 1786 W, respectively, and the joint TS1 and TP2 are not more than 200 W. However, the powers of the joint SY1 and SY2 in the whole process are completely equal because the structural feature of symmetrical leg and the connecting rods on the left and right sides are completely symmetrical, so their torques and speeds are also equal. The peak powers of the two joints of the symmetrical leg are approximately 845 W, which is approximately half of the peak powers of the other two legs.

As shown in Figure 2, in fact, the sum of the two joints’ power are almost equal, but the difference in the driving power of the two joints of TS and TP legs are relatively large, resulting in a large difference in the volume and weight of the two joints. It is difficult to achieve the symmetrical design and the repeated application of the joint module. However, the power of the two joints of the SY leg are exactly the same, which can reduce the design task. The result is that it can ensure the structural symmetry and dynamic balance, which is very important in dynamic movements, such as vertical jumping. Therefore, SY legs were selected to configure our quadruped robot.

### 2.2. Quadruped Robot with Manipulator

**Figure 3:**
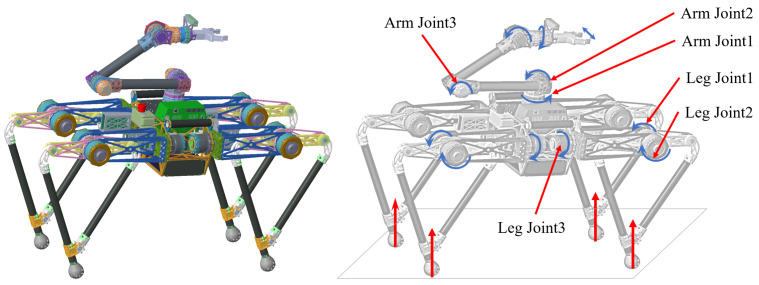
The SY legs quadruped robot with a manipulator.

Taking the torque and speed into account, a self-developed motor was selected to drive the joints, which has the top level in the same specification. Furthermore, a planetary reducer with a reduction ratio of 17:1 was selected by considering both the output torque and transparency during load movement. After calibrating the torque characteristics of the joint, it can achieve more accurate torque control and better reverse drive performance. The torso height (leg unfolded) and length of the robot are 80 cm and 120 cm, respectively. As shown in Figure 3, the total robot mass (including all battery and controllers) is approximately 35 kg, and each leg has three DoFs: the hip abduction/adduction (HAA) and two hip flexion/extension (HFE) configured in parallel. The weight of each single leg is mainly concentrated in the hip, and the mass ratio of the leg bar to the whole robot is less than 0.16. Our self-developed quadruped robot has achieved trotting, bounding and pronking gaits and has strong dynamic movement abilities.

The manipulator was attached to the front-middle of the quadruped robot [12,13], which can be used as the fifth leg or to remove debris from the ground, and the workspace is in front of the robot in this configuration. In this paper, the manipulator was designed, which has six active driving joints (including gripper driving) tailored for our quadruped robot, as shown in Figure 3. Furthermore, the whole mass of the manipulator is about 8 kg, as its compact in design and light in weight, and the mass of ME is about 3 kg. The manipulator was connected to the middle of the upper surface of the robot’s torso similar to a backward elbow. The advantage is that the manipulator weight is shared by four front legs, and the manipulator has a large movement space above the robot, which can realize the object movement and operation tasks on the desktop.

## 3. Bio-Inspired Take-Off Maneuver

In the process of the dynamic movement, such as jumping or running, a human always use their arm swing to assist movement and maintain body balance. Many researchers have explored the working principle of the arm swing from bionic kinematics. Hara [21] quantified and compared how the difference of arm-swing direction affects the mechanical and physiological parameters during jumping. Humanoid jumping and landing motion were analyzed from the perspective of the dynamic coupling of arm and jumping [22]. The conclusion was that due to the interference of the arm swing, the normal force rises and the time of leaving the ground is delayed, so the impulse and motion energy rise and the jumping height increased. Adrian [23] analyzed that the increased velocity of take-off stemmed from a complex series of events, which allowed the arms to build up energy early in the jump and transfer it to the rest of the body during the later stages of the jump. The jumping height of the robot was improved by effectively using the arm-swing motion and changing the foot posture [24]. The results showed that the jump height obtained is almost four times that obtained without arm swing.

**Figure 4:**
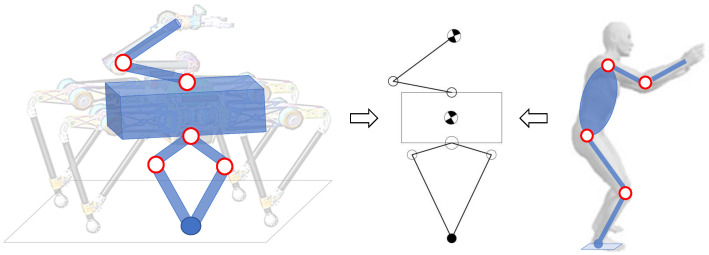
The simplified model of quadruped robot inspired by human jumping.

According to the existing research on human jumping, it is found that the arm-swing direction and trigger time have significant important impact on the jumping performance. Inspired by the arm-swing assisted jumping of human, we simplified the quadruped robot with a manipulator into a human-like upper and lower limbs model. Since the phases of the four legs are consistent in the process of vertical jumping, it can be simplified into a virtual single leg directly below the torso, as shown in Figure 4. Compared with the human lateral model, the simplified single leg is in the form of a symmetrical connecting rod that is parallel-driven, which has better jumping ability according to the analysis in Section 2. The simplified arm is a two-link model installed directly above the torso, which corresponds to the simplified two-link model of the human arm. It should be noted that the model in Figure 4 is only simplified to analyze the inspired jumping strategy (motion sequence). All joints of the four legs and the manipulator are active and controllable in the later motion tracking.

**Figure 5:**
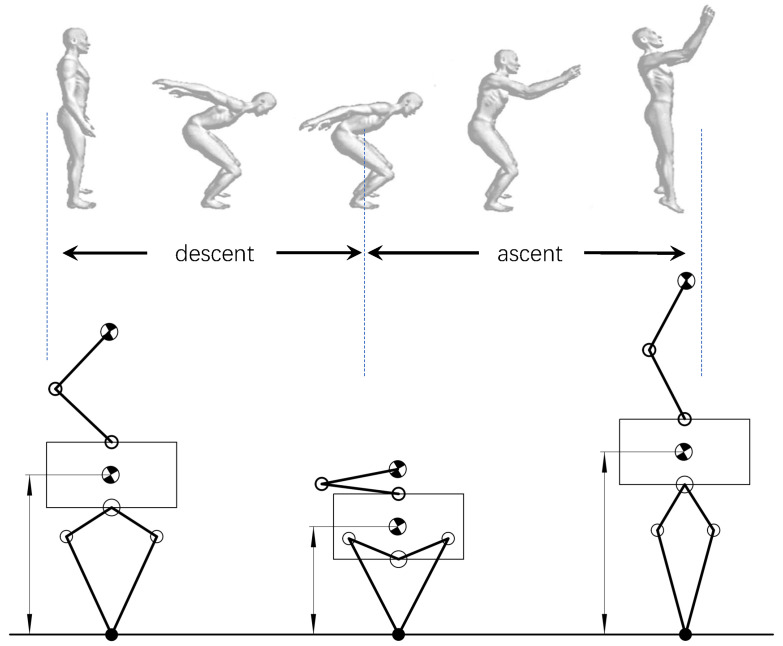
The decomposition motion during vertical jumping.

Then, we analyzed the decomposition of human upper and lower limbs movement in the whole process of vertical jumping. As shown in the image above Figure 5, in the descent stage, the experimenter squatted from the natural standing state to the initial position of jumping, and the arm swings back from the natural drooping. In the ascent stage, the arm swings counterclockwise around the shoulder joint during the rise of the torso. At the moment of leaving the ground, the leg extends to the maximum length, and the end of the arm swings above the shoulder joint.

Based on the above human vertical jump decomposed action, we proposed a bio-inspired take-off maneuver based on the coordination of upper and lower limbs for the quadruped robot with a manipulator. As shown in the image below Figure 5, the quadruped robot with a manipulator squats to the initial height of jump to achieve greater upward acceleration distance. In the take-off stage, the kinetic energy and potential energy of the system are increased by driving the ME to swing upward, and the center of mass (CoM) of the system will rise. The downward thrust generated by the ME in the accelerated rising process is the load of the lower limbs, so the upper limbs will reach the peak speed and start decelerating in advance during the same movement cycle of the upper and lower limbs. In the deceleration process of ME, it will produce an upward pull force on the body, which will reduce the load of the lower limbs at the peak speed, thus reducing the peak powers demand of legs’ joints. Therefore, the jumping ability of the system is improved.

It is worth noting that in the ascent stage, the arm-swing direction is along the vertical direction, which can ensure that there is only additional downward force perpendicular to the torso to maintain body balance. The proposed method of the arm-swing motion can be applied to most movement modes. For different jumping movements, the expected effect can be produced by changing the direction of the arm swing. It is similar to the forward swing of arms in human long jump, the long-jump performance can be increased by the forward swing of the arm instead of the upward swing in the vertical jump process. Moreover, the trigger time of the arm swing affects the jump height, and it was optimized in the simulation part.

## 4. Motion Tracking Based on Whole-Body Control

**Figure 6:**
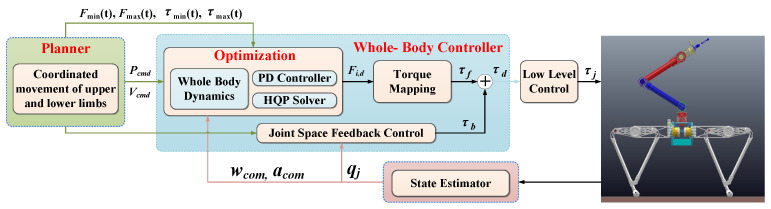
Locomotion framework based on whole-body controller.

This section introduces our control framework, which includes locomotion planner, whole-body controller, state estimator and a low-level torque controller, as shown in Figure 6. The control tasks are tracking the position and the orientation of the robot base, and it can be achieved through calculating the ground reaction forces (GRFs) at the standing phase to generate the required acceleration and the angular acceleration of the robot base. Considering the constraints of the whole-body dynamics model, friction cone and joint driving ability, the optimal contact force of each iteration of the control loop is solved using the hierarchical optimization method. Then the optimal solution is mapped into joint torque and sent to the low-order torque controller.

### 4.1. Model Formulation

The quadruped robot with manipulator is a highly coupled multi-link system with four legs distributed in parallel and connected in series with a manipulator, resulting in a large amount of calculation in the process of dynamic modeling and optimization. According to the analysis in Section 2, the leg mass of our robot is very small. In order to facilitate our calculation, we simplified the robot into a floating single rigid body except for the manipulator, which is connected with the inertial coordinate system through the virtual six-dimensional force. Therefore, the whole system is simplified into a floating seven-link system, which has six virtual driving joints connected to the inertial system (three translational DoFs and three rotational DoFs) and six active joints from the manipulator.

The motion of the whole system can be described in a fixed inertial frame *I*. The torso position and base orientation are written as $r_{IB}\in R^{3}$, $q_{IB}\in SO\left(3\right)$, respectively. The joint angles of the manipulator are stacked in the vector $q_{j}\in R^{n_{j}}$, where $n_{j}$ = 6. Then, the system generalized coordinate vector q and the generalized velocity vector $\dot{q}$ can be written as:

(4) $$q=\left[\begin{array}{c} r_{IB}\\ q_{IB}\\ q_{j} \end{array}\right]\in SE\left(3\right)\times R^{n_{j}},\;\dot{q}=\left[\begin{array}{c} v_{IB}\\ \omega_{IB}\\ {\dot{q}}_{j} \end{array}\right]\in R^{n_{u}}$$

where $n_{u}$ = 6 + $n_{j}$, and $v_{IB}$ and $\omega_{IB}$ are the linear and angular velocity of the base, respectively. The equation of motion of the floating base system interacting with the environment is written as:

(5) $$\left[\begin{array}{cc} M(q) & -S \end{array}\right]\left[\begin{array}{c} \ddot{q}\\ T \end{array}\right]+h\left(q,\dot{q}\right)=0,$$

(6) $$T={\left[\begin{array}{ccc} F_{s}^{3\times 1} & M_{s}^{3\times 1} & \tau_{j}^{6\times 1} \end{array}\right]}^{T}$$

where $M(q)\in R^{n_{u}\times n_{u}}$ is the inertia matrix, $h(q,\dot{q})\in R^{n_{u}}$ is the sum of gravity, centrifugal and Coriolis forces, S is a selection matrix that selects which joints are actuated, $F_{s}$ and $M_{s}$ represent the virtual three-dimensional force and three-dimensional moment of the system from the ground, respectively.

### 4.2. Whole-Body Control Based on Hierarchical Optimization

#### 4.2.1. Hierarchical Optimization

Traditional quadratic programming (QP) achieves the optimization of target motion through setting the corresponding weight coefficients according to different task priorities. This method is difficult to achieve the absolute priority between different tasks. In this paper, hierarchical optimization is used to solve a series of QP problems according to priority by gradually reducing the solution space. A task or tasks with the same priority W can be defined as a set of linear equality and inequality constraints on the solution vector X:

(7) $$W\;:\;\left\{\begin{array}{c} A_{eq}X-b_{eq}=\varepsilon \\ A_{ieq}X-b_{ieq}\leq v \end{array}\right.$$

where X is the optimization objective, and ε and v are the relaxation variables to be minimized. $A_{eq}$, $b_{eq}$, $A_{ieq}$, and $b_{ieq}$ represent the coefficient matrix of equality constraints and inequality constraints, respectively.

An optimal solution $X^{*}$ can be obtained by solving *n* tasks according to the specified priority order. In order to ensure strict priority, the next solution $X_{n+1}$ is found in the null space $Z_{n}=N{\left(A_{eq1}^{T},A_{eq2}^{T}\ldots A_{eqn}^{T}\right)}^{T}$ with equal constraints of all higher priorities, $X_{n+1}=X_{n}^{*}+Z_{n}z_{n+1}$, where $z_{n+1}$ is a vector that lives in the row space of $Z_{n}$ [25].

(8) $$\begin{array}{cc} \min_{z_{n+1},v_{n+1}} & {∥A_{eq(n+1)}\left(X^{*}+Z_{n}z_{n+1}\right)-b_{eq(n+1)}∥}^{2}+{∥v_{n+1}∥}^{2}\\ \;s.\;t.\; & {A_{ieq}}_{\left(n+1\right)}\left(X^{*}+Z_{n}z_{n+1}\right)-{b_{ieq}}_{\left(n+1\right)}\leq v_{n+1}\\ & {A_{ieq}}_{\left(n\right)}\left(X^{*}+Z_{n-1}z_{n}\right)-{b_{ieq}}_{\left(n\right)}\leq v_{n}\\ \; & \vdots \\ & {A_{ieq}}_{\left(1\right)}X^{*}-{b_{ieq}}_{\left(1\right)}\leq v_{1}\\ & v_{n+1}⩾0. \end{array}$$

The optimization problem is solved from high to low priority, and the low-priority solution is searched in the null space of the high-priority solution. Because more equations are added to the task stack, the iterative method is used to solve. As the priority decreases, the size of the null space will also decrease, so the calculation speed will become faster.

#### 4.2.2. Task Formulation

According to the motion framework shown in Figure 6, we first give the motion trajectory in the operational space for the specific components of the system (manipulator and torso) in the advanced controller, including the position of the torso, the direction of the base and the position and attitude of the manipulator. In this paper, the cubic spline interpolation method is used to generate the trajectory, which can ensure that the starting and ending velocities and accelerations are 0. Then, the expected force is optimized by the whole-body controller. We express the WBC problem as a QP problem composed of linear equality and inequality tasks, such as the form in Equation (8). Then, the hierarchical optimization algorithm is used to solve the optimization problem.

According to Equations (5) and (6), the optimization objective of the simplified seven-link model can be uniformly written as:

(9) $$X={\left[\begin{array}{cc} {\ddot{q}}^{T} & T^{T} \end{array}\right]}^{T}$$

The system dynamics model represented by Equation (5) is an imposed equality constraint, which makes the task with the highest priority. In addition, inequality constraints need to be added, including joint torque limit and contact force limit caused by friction cone:

(10) $$\left\{\begin{array}{c} \tau_{\min}\leq \tau \leq \tau_{\max}\\ -uF_{z}\leq F_{x/y}\leq uF_{z} \end{array}\right.$$

where *u* is the friction coefficient of the contact surface.

The most important point in motion tracking is that we should define the corresponding constraints according to the desired motion. For the motion characteristics in the vertical jumping, the task constraints in this paper mainly include:

- *Contact constraint:* It is a very important condition to ensure that the support point does not slide with the ground in the support phase of the legged robot, which can be achieved by constraining the target acceleration:
  (11) $$I_{24}X=\left[\begin{array}{c} {\ddot{q}}_{torso\_d}^{6\times 1}\\ 0_{18\times 1} \end{array}\right]$$
  where the ${\ddot{q}}_{torso\_d}^{6\times 1}$ is the desired acceleration vector of the torso obtained from the planner.
- *Motion tracking in operational space:* The control goal is to achieve accurate motion tracking and make the system move along the desired trajectory. According to Section 3, it is necessary to ensure the motion of the upper and lower limbs is at the same time in the jumping motion planning. For the pre-planned torso and manipulator trajectory (position, velocity and acceleration), as shown in Figure 6, the motion tracking in the operational space can be realized by the following constraints:
  (12) $$\left[\begin{array}{ccc} I_{6} & J_{arm}^{6\times 6} & I_{12} \end{array}\right]X=\left[\begin{array}{c} {\ddot{q}}_{torso\_d,p}^{3\times 1}+K_{p,p}\left(p_{torso\_d}-p_{torso}\right)+K_{d,p}\left({\dot{p}}_{torso\_d}-{\dot{p}}_{torso}\right)\\ {\ddot{q}}_{torso\_d,\omega }^{3\times 1}+K_{p,\omega }\log\left(R_{d}R^{T}\right)+K_{d,\omega }\left(\omega_{torso\_d}-\omega_{torso}\right)\\ {\ddot{q}}_{arm\_d}^{6\times 1}-{\dot{J}}_{arm}^{6\times 6}{\dot{q}}_{arm}\\ 0_{12} \end{array}\right]$$
  where the $J_{arm}^{6\times 6}$ is the Jacobian of the arm-end motion. The desired acceleration $\ddot{q}$ is solved by PD control law and can be divide into three parts: torso position, base orientation and manipulator’s position. Furthermore, the orientation error is obtained using the exponential map representation of rotations, where $R_{d}$ and *R* represent the desired and actual base orientation, respectively.
- *Energy optimization:* Similar to the optimization goal of motion tracking, we can also introduce the task of driving force or torque. These tasks usually have the lowest priority, and it is usually used to improve the energy efficiency of the system:
  (13) $$\left[\begin{array}{cc} 0_{12} & I_{12} \end{array}\right]X=0$$

The above task constraints mainly aim at the constraints necessary in the jump motion. Table 1 gives the task priority order specified in our experiment. The task with the highest priority abides by the dynamic model, physical constraint of actuator and motion assumptions, such as friction cone constraint and no sliding. The second is to ensure the desired motion tracking. Finally, the energy efficiency of the system is improved by optimizing the driving torque. In addition, we can add swing leg and ZMP tracking optimization during walk, trot and other movements to achieve more accurate and stable locomotion and increase the manipulable optimization in the manipulator’s operation tasks.

### 4.3. Joint Torque Generation

The simplified dynamic model of the system is described above, as shown in Equation (5). The simplified model combined with the planned trajectory and motion optimization based on hierarchical optimization obtains the optimized target variables X, which includes the six-dimensional force acting on the torso and the torque of the six joints from the manipulator, and the latter can be used as the input of low-level control directly. At the same time, we need to map the virtual six-dimensional force ${\left[F_{s},M_{s}\right]}^{T}$ acting on the torso in the operational space to the joint space of the robot’s legs. Firstly, we obtain the optimal distribution of GRFs through Equations (14) and (15), then map the force to the corresponding joint through the leg’s Jacobian matrix $J_{leg}$.

(14) $$\underset{A}{\underset{︸}{\left[\begin{array}{c} \begin{array}{ccc} I_{3} & \cdots & I_{3} \end{array}\\ \begin{array}{ccc} r_{1}\times & \cdots & r_{i}\times \end{array} \end{array}\right]}}\underset{f}{\underset{︸}{\left[\begin{array}{c} F_{\mathrm{leg},1}\\ \ldots \\ F_{\mathrm{leg},i} \end{array}\right]}}=\underset{b}{\underset{︸}{\left[\begin{array}{c} F_{s}\\ M_{s} \end{array}\right]}},$$

where $r_{i}\times \in R^{3\times 3}$ and $F_{leg,i}$ represent the relative position matrix and the GRF of the *i*th leg, respectively.

According to Equation (14), the optimal force distribution can be transformed into an optimization problem that aims to find the minimum force as follows:

(15) $$\begin{array}{c} f^{d}=\underset{f\in R^{6}}{\arg\min}{\left(Af-b\right)}^{⊤}S\left(Af-b\right)+\alpha f^{⊤}Wf\;\\ \;s.t.\;-\mu F_{\mathrm{leg},i}^{n}\leq F_{\mathrm{leg},i}^{t}\leq \mu F_{\mathrm{leg},i}^{n} \end{array}$$

where $S,W\in R^{6\times 6}$ are the positive-definite weight matrices; α∈R is the secondary objective; μ is the dynamic friction coefficient of contact plane.

The above minimum problem can be transformed into a QP optimization problem for the convenience of calculation. To obtain the desired motion tracking, the GRFs of each supporting foot need to meet the basic physical constraints, including the friction cone constraint and the unidirectionality of the leg output force in *z* direction.

## 5. Numerical Simulations and Discussions

This section presents the simulations and results performed for the jumping motion of the quadruped robot with a manipulator based on the proposed methods, and the CoppeliaSim dynamic software was used to simulate the jumping motion. The Earth environment with the largest gravitational acceleration was chosen to verify the advantages of the take-off maneuver proposed in this paper. The physical parameters and simulation settings are shown in Table 2.

### 5.1. Trigger Time Optimization of Arm Swing

The cooperative take-off maneuver of upper and lower limbs based on arm swing was proposed by analyzing the mechanism of a human arm swing jump in Section 3. After adding the arm swing, the robot base squats down from the natural standing height $h_{sta}$ to the initial jumping height $h_{ijb}$, and the ME moves to the initial position $h_{ijm}$. Then, the motion of the base and the ME are triggered at a pre-set time and move to $h_{lea}$ and the maximum position of ME $h_{mm}$, respectively. The robot will flight and maintain the state at leaving the ground. After the robot lands, the robot system with a manipulator should be adjusted to the initial position before take-off. This process is the coordinated movement of the whole system, including leg length recovery and manipulator recovery.

The trigger time and the speed of arm swing will directly affect the load and work characteristics of lower limbs, so as to affect the jumping height. It can be concluded that the faster the arm swings, the higher the jump height is when the driving conditions are available through a series of experiments of different swing cycles. The reason is that the higher swing speed will lead to greater load and increase the work of leg joints. Delayed arm swing was only studied in [22], we compared the affect of trigger time through a series of experiments on the premise that the trajectory of the torso and arm remains unchanged. We set seven arm swing trigger times: − 0.02 s, −0.04 s, −0.06 s, 0, 0.02 s, 0.04 s and 0.06 s, where the negatives represent arm starts swinging ahead of the base, 0 represents the simultaneous trigger motion, the positives represent arm delayed swinging.

In the above comparative experiments, the cycles of torso rising and arm swing are equal $(T_{j}=0.14$ s). The measured maximum values of torso height corresponding to different arm trigger times are shown in Table 3; we can concluded that the jump height reaches the maximum when the upper and lower limbs motion were triggered simultaneously. Combining the above analysis of motion mechanism, the downward thrust generated by the ME in the accelerated rising process is the load of the lower limbs, so the ME will reach the peak speed and start decelerating in advance during the same movement cycle of the upper and lower limbs. However, when the arm swings in advance, the cross area between the deceleration time of ME and the peak stage of lower limb power decreases, and the pull efficiency from the ME cannot be maximized. When the arm is delayed swing, the ME may reach the peak speed and start decelerating with the lower limbs at the same time or delaying, so it will not reduce the peak power demand of the lower limbs. Therefore, only when the movements of the upper and lower limbs are triggered at the same time can the cross area between the ME deceleration stage and the lower limb power peak stage be increased, which will reduce the load of the lower limbs at the peak speed and reduce the demand for their peak powers.

### 5.2. Comparison of Vertical Jumping with or without Arm Swing

**Figure 7:**
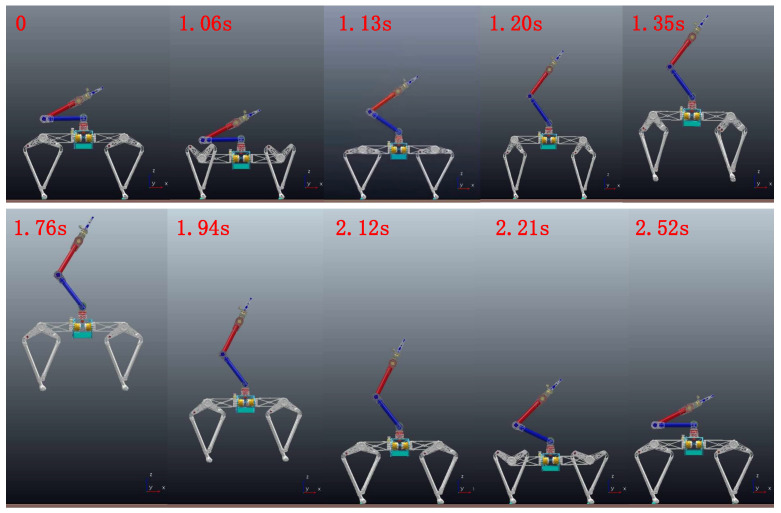
The snapshot of the single vertical jumping process.

In order to verify the improvement of arm swing on jump performance, we analyze it by comparing the net jump height Δh and peak power of each leg joint with or without arm swing on the Earth. In this group of comparative experiments, all other parameters and initial conditions are exactly the same except the arm-swing movement in the take-off stage. The snapshot of the entire vertical jumping process is shown in Figure 7.

**Figure 8:**
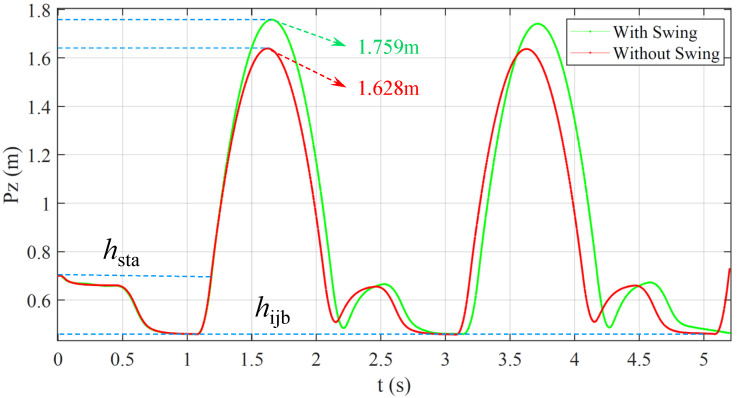
The height change curve of robot torso during jumping process.

The actual net jumping height of torso Δh is the difference between the maximum jumping height $h_{max}$ and $h_{lea}$. It can be seen from Figure 8 that the maximum values of torso height are 1.759 m and 1.628 m, respectively. The net jump heights Δh are 1.009 m and 0.878 m, respectively, with and without arm swing. In fact, the highest point of ME relative to torso is $h_{mm}$ when added the arm swing, and this value is $h_{ijm}$ when there is no arm swing, so the relative height of ME is: $\Delta h_{me}=\frac{M_{me}}{M_{s}}\left(h_{mm}-h_{ijm}\right)$. Furthermore, the jumping height of the whole CoM is: $\Delta h+\Delta h_{me}$. Therefore, the jump height increases by 19% after adding arm swing.

**Figure 9:**
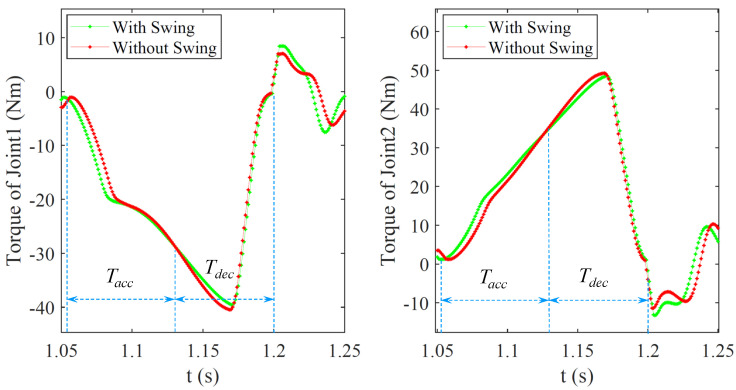
The joint torques the robot leg during the take-off phase.

**Figure 10:**
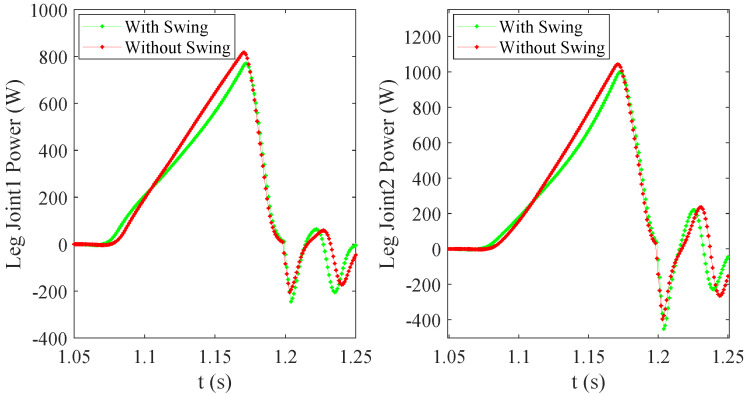
The joint powers of the robot leg during the take-off phase.

In order to further analyze the impact of arm swing on jumping performance, we analyzed the torque and power of the key leg Joint1 and leg Joint2, as shown in Figure 3. The curves in the take-off phase were analyzed to make a clearer representation, and the take-off stage (0.14 s) was divided into acceleration stage $(T_{acc}$: 1.06–1.13 s) and deceleration stage $(T_{dec}$: 1.13–1.2 s). The dividing point is 1.13 s, where the upward acceleration of the ME is 0. The torque change of the leg joints during the jumping process is shown in Figure 9. The leg joint torques in $T_{acc}$ are greater than that without swing because the leg needs to overcome the reverse thrust from the upper limb. However, the ME generates upward pull force on the torso in $T_{dec}$, and the leg joint torques are less than that without swing, which verifies the theory in Section 3. At the same time, the joint output powers calculated according to Equation (3) were compared, as shown in Figure 10. The peak powers of leg Joint1 are 770 W and 816 W, and the peak powers of leg Joint2 are 998 W and 1043 W with and without arm swing, respectively. Due to the upward pull of ME during the deceleration phase, the leg’s load is reduced, so the required peak power is reduced. In addition, the peak powers of Joint1 and Joint2 are not equal, which is due to the additional supporting foot parts in the structural realization of symmetrical legs, as shown in Figure 7, resulting in incomplete symmetry in the leg structure.

**Figure 11:**
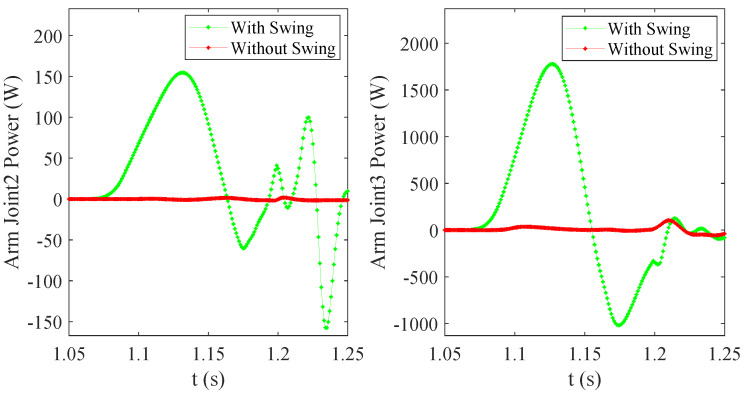
The joint powers of the manipulator during the take-off phase.

We also analyzed the power of the key Joint2 and Joint3 from arm in the jumping process with arm swing, as shown in Figure 3. As shown in Figure 11, the peak powers of arm Joint2 and Joint3 are 157 W and 1776 W, respectively. The peak power of Joint3 is larger, and the characteristics of this joint are larger torque and relatively smaller speed, which can be realized by selecting a high reduction ratio driving module. Furthermore, its characteristics meet the joint performance requirements of the manipulator operation tasks.

In order to explore the performance of the take-off maneuver proposed on the other planets, we conducted jumping simulations in the Moon and the Mars environments (only considering the impact from gravitational acceleration), which humans have landed and explored. Under the same motion planning (on flat ground), the jump heights and the actual data of each joint in the two environments were analyzed, as shown in the fourth and fifth rows of Table 4. It can be found that the jump height increments with arm swing are 17.1% and 15.4% in the Mars and the Moon, respectively. The peak powers of the two joints of the leg are all less than the values without arm swing.

**Figure 12:**
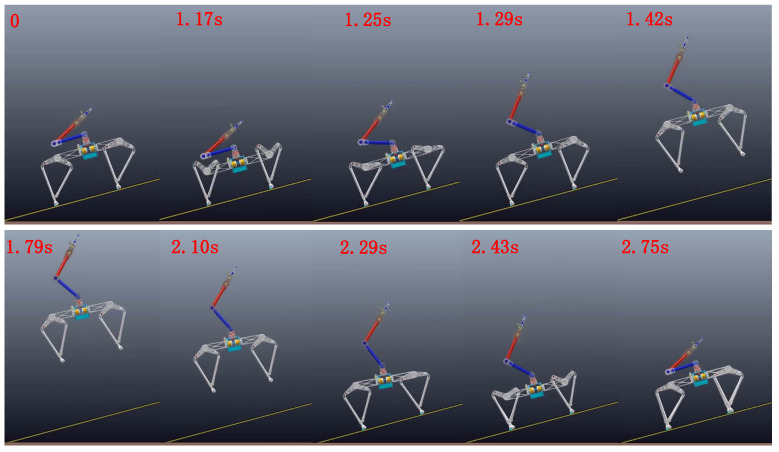
The snapshot of the jumping with arm swing on 15° slope.

Moreover, in order to explore the performance of the take-off maneuver and control method proposed in this paper on the inclined planes, we chose to perform a comparative jump simulation on a 15° slope on the Earth’s surface. To achieve continuous and stable jumping on the slope, the robot needs to adapt the body to the slope during the take-off phase and before landing. This adaptive balance strategy is unified for take-off on flat ground (the angle of slope is 0). Except for the leaving height $h_{lea}$ (the height of the robot torso on the slope in the world coordinate system is 0.95 m), the other parameters are same as those in Table 2. The continuous and stable jumping on the 15° slope was realized using the take-off maneuver and control method proposed in this paper, and the snapshot of the jumping on a 15° slope is shown in Figure 12. According to the data in the last row of Table 4, the jump height increments after adding arm swing is 17.4%; the peak power of leg Joint1 and Joint2 are 802 W and 922 W, respectively, which is lower than the values without arm swing. Compared with the jumping on the Earth flat ground, the jump height increment on the slope is lower. This is mainly because it needs to ensure the system is balanced on the slope during the take-off phase, which restricts the motion tracking of the arm (the peak power of arm Joint3 is lower than the value on flat ground). Furthermore, it can be improved by a more accurate system model and dynamic parameter calibration improvement. We will further explore the jump control on the slope in future research. It can also be found that the peak power of leg Joint1 on a 15° slope is larger than the value on flat ground, and the peak power of Joint2 is lower. The reason is that the legs need to rotate at the corresponding angle around the hip joint to ensure the balance of robot system on the incline, which leads to the leg rods driven by Joint1 and Joint2 to rotate counterclockwise. Thus, the leg rotation reduces the asymmetry caused by the foot structure and reduces the difference between the peak power that the two leg joints need to provide larger torque.

According to the above simulation results and discussions, the peak powers required by the leg joints are reduced and the jump height improved after adding arm swing in different gravity environments and 15° slope, as shown in Table 4. The superiority of the take-off maneuver proposed in this paper and the stability of jump control are verified.

## 6. Conclusions

In this paper, we showed how to make rational use of the active DoFs of a manipulator to overcome the challenge of jumping for a quadruped robot with a manipulator. Based on our previous research, the peak powers requirement of the joints from different leg types in the jumping motion were further analyzed, and the symmetrical leg with the lowest power requirements and superior joint manipulability was selected to configure our robot. At the same time, a manipulator with six DoFs was tailored for our quadruped robot.

In order to improve the jumping height of the quadruped robot with manipulator, a cooperative jumping maneuver of the upper and lower limbs inspired by human arm-swing jumping was proposed. In the take-off stage, the kinetic energy and potential energy of the system are increased by driving the ME to swing upward, and the CoM of the system will rise. Because the downward thrust generated by the ME in the accelerated rising process is the load of the lower limbs, the upper limbs will reach the peak speed and start decelerating in advance during the same movement cycle of the upper and lower limbs. In the deceleration process of ME, it will produce an upward pull force on the body, which will reduce the load of the lower limbs at the peak speed, thus reducing the peak power demand of legs’ joints. Therefore, the jumping ability of the system is improved. Furthermore, the optimal motion planning was obtained through comparing the influence of the trigger time of upper and lower limbs on the jumping height. At the same time, a motion tracking method was proposed to achieve stable jumping, in which the quadruped robot with a manipulator is simplified to a floating seven-link model. The desired virtual six-dimensional force acting on the base (robot torso and four legs) and the torque acting on the manipulator are solved based on WBC and the hierarchical optimization method. Then, the optimal GRFs calculated by QP are mapped to the corresponding joint space. This method greatly simplifies the dynamic model and is convenient for calculation. Finally, the jumping simulations in different gravity environments and a 15° slope were performed. The jump heights were all improved after adding the arm swing, and the peak powers of leg joints are smaller. Thus, the superiority of the coordinated take-off maneuvers of the upper and lower limbs proposed in this paper were verified. Furthermore, the stability of the jumping control method was testified by the continuous and stable jumping.

In future work, we will explore the maneuver of forward jumping and the attitude control in flight-phase; realize stable jumping and robust landing in complex terrain; and further improve the dynamic motion ability of quadruped robot with manipulator. 

## Figures and Tables

**Table 1:** The describe of task priority in experiments.

Priority	Task
1	Equations of Motion
	No contact motion
	Torque limits
	Friction cone limits
2	Torso position tracking
	Torso orientation tracking
	Manipulator motion tracking
3	Contact force minimization
	Joint torque minimization

**Table 2:** Parameters for simulation and experiment.

Symbol	Items	Values
$M_{s}$	Total mass	43 kg
$M_{sl}$	Single leg mass	1.4 kg
$M_{m}$	Manipulator mass	8 kg
$M_{me}$	Manipulator-end mass	3 kg
$L_{body}$	Length of robot	1.2 m
$W_{body}$	Width of robot	0.4 m
$L_{m}$	Length of Manipulator	0.76 m
$T_{j}$	Jump period	0.14 s
Step	Control period	1 ms
$h_{sta}$	Stance height	0.7 m
$h_{lea}$	Leaving height	0.75 m
$h_{ijb}$	Initial height of base	0.45 m
$h_{mm}$	Maximum height of manipulator-end	0.7 m
$h_{ijm}$	Initial height of manipulator-end	0.2 m
$g_{Earth}$	Earth’s gravitational acceleration	9.8 m/s${}^{2}$

**Table 3:** The maximum height of torso with different trigger times.

$T_{tri}$(s)	−0.06	−0.04	−0.02	0	0.02	0.04	0.06
$h_{max}$(m)	1.724	1.738	1.751	1.759	1.757	1.729	1.676

**Table 4:** The comparison of jumping performance with or without arm swing in different environments. ($P_{lj1},P_{lj2},P_{aj2}$ and $P_{aj3}$ are the peak power of leg Joint1, leg Joint2, arm Joint2 and arm Joint3, respectively. ΔH represents the percentage of jump height increments.)

Environment	*g* (m/s${}^{2}$)	Condition	$P_{lj1}$ (W)	$P_{lj2}$ (W)	$P_{aj2}$ (W)	$P_{aj3}$ (W)	$h_{max}$ (m)	ΔH(%)
Earth (flat ground)	9.8	with swing	770	998	157	1776	1.759	19
		without swing	816	1043	0	0	1.628	
Mars (flat ground)	3.71	with swing	848	1028	140	1830	3.766	17.1
		without swing	903	1104	0	0	3.405	
Moon (flat ground)	1.63	with swing	778	971	159	1862	7.632	15.4
		without swing	928	1106	0	0	6.9	
Earth (15° slope)	9.8	with swing	802	922	287	1460	1.873	17.4
		without swing	860	983	0	0	1.764	
